# 2,3-Bis(pyrazin-2-yloxyimino)butane

**DOI:** 10.1107/S1600536808029991

**Published:** 2008-09-24

**Authors:** Lin Yan Yang, Jing Min Shi

**Affiliations:** a Department of Chemistry, Shandong Normal University, Jinan 250014, People’s Republic of China

## Abstract

The title mol­ecule, C_12_H_12_N_6_O_2_, lies on a crystallographic inversion center with all non-H atoms essentially coplanar.

## Related literature

For a related structure, see: Chen & Yang (2008 ▶).
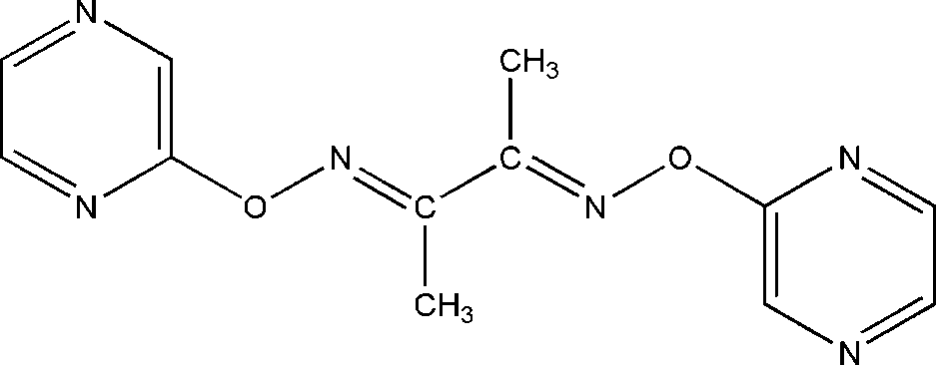


## Experimental

### 

#### Crystal data

C_12_H_12_N_6_O_2_
*M* *_r_* = 272.28
Monoclinic, 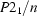

*a* = 4.7396 (15) Å
*b* = 17.141 (5) Å
*c* = 7.911 (3) Å
β = 98.065 (5)°
*V* = 636.3 (4) Å^3^
*Z* = 2
Mo *K*α radiation
μ = 0.10 mm^−1^
*T* = 298 (2) K
0.43 × 0.10 × 0.06 mm

#### Data collection

Bruker SMART APEX CCD diffractometer
Absorption correction: multi-scan (*SADABS*; Sheldrick, 1996 ▶) *T*_min_ = 0.957, *T*_max_ = 0.994
3621 measured reflections
1368 independent reflections
872 reflections with *I* > 2σ(*I*)
*R*_int_ = 0.036

#### Refinement

*R*[*F* ^2^ > 2σ(*F* ^2^)] = 0.057
*wR*(*F* ^2^) = 0.142
*S* = 1.03
1368 reflections
92 parameters
H-atom parameters constrained
Δρ_max_ = 0.19 e Å^−3^
Δρ_min_ = −0.16 e Å^−3^

### 

Data collection: *SMART* (Bruker, 2007 ▶); cell refinement: *SAINT* (Bruker, 2007 ▶); data reduction: *SAINT*; program(s) used to solve structure: *SHELXTL* (Sheldrick, 2008 ▶); program(s) used to refine structure: *SHELXTL*; molecular graphics: *SHELXTL*; software used to prepare material for publication: *SHELXTL*.

## Supplementary Material

Crystal structure: contains datablocks I, global. DOI: 10.1107/S1600536808029991/lh2694sup1.cif

Structure factors: contains datablocks I. DOI: 10.1107/S1600536808029991/lh2694Isup2.hkl

Additional supplementary materials:  crystallographic information; 3D view; checkCIF report

## Figures and Tables

**Fig. 1.**
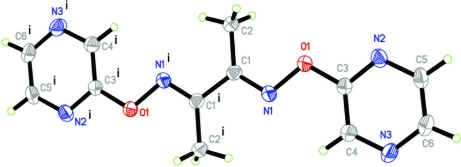
Molecular structure of the title compound with the atom-numbering scheme. Displacement ellipsoids are drawn at the 30% probability level [symmetry code: (i) -x+1, -y+1, -z].

## supplementary crystallographic information

### Comment

We are interested in  the design and synthesis of muliti-dentate ligands containing pyrazinyl and butane-2,3-dione dioxime and  hence we have previously synthesized (2E,3E)-3-(pyrazin-2-yloxyimino)butane-2-one oxime (Chen *et al.*, 2008) and now we report herein  homologous title compound (I).

Fig. 1 shows the molecular structure with an inversion centre located in the middle of the C1-C1^i^ bond [symmetry code: (i) -x+1, -y+1, -z]. All of the non-hydrogen atoms define a plane within 0.0652 Å with a maximum deviation of 0.1212 (16) Å for atom C4.

### Experimental

A powder of the title compound (0.0473 g, 0.174 mmol) was dissolved into a mixture of solvents containing 20 ml dichloromethane and 10 ml methanol. The colorless single crystals were obtained after the solution was allowed to stand at room temperature for two days.

### Refinement

All H atoms were placed in calculated positions and refined as riding with C—H = 0.96 Å, *U*_iso_ = 1.5*U*_eq_(C) for methyl group and C—H = 0.93 Å, *U*_iso_ = 1.2*U*_eq_(C) for pyrazinyl H atoms.

### Crystal data

**Table:**

C_12_H_12_N_6_O_2_	*F*(000) = 284
*M**_r_* = 272.28	*D*_x_ = 1.421 Mg m^−^^3^
Monoclinic, *P*2_1_/*n*	Mo *K*α radiation, λ = 0.71073 Å
Hall symbol: -P 2yn	Cell parameters from 556 reflections
*a* = 4.7396 (15) Å	θ = 2.4–20.6°
*b* = 17.141 (5) Å	µ = 0.10 mm^−^^1^
*c* = 7.911 (3) Å	*T* = 298 K
β = 98.065 (5)°	Needle, colorless
*V* = 636.3 (4) Å^3^	0.43 × 0.10 × 0.06 mm
*Z* = 2	

### Data collection

**Table:**

Bruker SMART APEX CCD diffractometer	1368 independent reflections
Radiation source: fine-focus sealed tube	872 reflections with *I* > 2σ(*I*)
graphite	*R*_int_ = 0.036
φ and ω scans	θ_max_ = 27.0°, θ_min_ = 2.4°
Absorption correction: multi-scan (SADABS; Sheldrick, 1996)	*h* = −6→5
*T*_min_ = 0.957, *T*_max_ = 0.994	*k* = −21→17
3621 measured reflections	*l* = −9→10

### Refinement

**Table:**

Refinement on *F*^2^	Primary atom site location: structure-invariant direct methods
Least-squares matrix: full	Secondary atom site location: difference Fourier map
*R*[*F*^2^ > 2σ(*F*^2^)] = 0.057	Hydrogen site location: inferred from neighbouring sites
*wR*(*F*^2^) = 0.142	H-atom parameters constrained
*S* = 1.03	*w* = 1/[σ^2^(*F*_o_^2^) + (0.062*P*)^2^ + 0.0016*P*] where *P* = (*F*_o_^2^ + 2*F*_c_^2^)/3
1368 reflections	(Δ/σ)_max_ < 0.001
92 parameters	Δρ_max_ = 0.19 e Å^−^^3^
0 restraints	Δρ_min_ = −0.16 e Å^−^^3^

### Special details

**Table:**

Geometry. All e.s.d.'s (except the e.s.d. in the dihedral angle between two l.s. planes) are estimated using the full covariance matrix. The cell e.s.d.'s are taken into account individually in the estimation of e.s.d.'s in distances, angles and torsion angles; correlations between e.s.d.'s in cell parameters are only used when they are defined by crystal symmetry. An approximate (isotropic) treatment of cell e.s.d.'s is used for estimating e.s.d.'s involving l.s. planes.
Refinement. Refinement of *F*^2^ against ALL reflections. The weighted *R*-factor *wR* and goodness of fit *S* are based on *F*^2^, conventional *R*-factors *R* are based on *F*, with *F* set to zero for negative *F*^2^. The threshold expression of *F*^2^ > σ(*F*^2^) is used only for calculating *R*-factors(gt) *etc*. and is not relevant to the choice of reflections for refinement. *R*-factors based on *F*^2^ are statistically about twice as large as those based on *F*, and *R*- factors based on ALL data will be even larger.

### Fractional atomic coordinates and isotropic or equivalent isotropic displacement parameters (Å^2^)

**Table:**

	*x*	*y*	*z*	*U*_iso_*/*U*_eq_	
C1	0.4107 (4)	0.53547 (11)	−0.0115 (3)	0.0380 (5)	
C2	0.4063 (5)	0.58565 (13)	−0.1667 (3)	0.0555 (7)	
H2A	0.2172	0.5864	−0.2290	0.083*	
H2B	0.5364	0.5650	−0.2380	0.083*	
H2C	0.4623	0.6378	−0.1326	0.083*	
C3	−0.0460 (5)	0.63257 (12)	0.2132 (3)	0.0378 (5)	
C4	−0.0315 (5)	0.58891 (14)	0.3610 (3)	0.0493 (6)	
H4	0.0872	0.5455	0.3752	0.059*	
C5	−0.3601 (5)	0.71330 (13)	0.3082 (3)	0.0507 (7)	
H5	−0.4777	0.7569	0.2942	0.061*	
C6	−0.3497 (5)	0.67050 (14)	0.4551 (3)	0.0550 (7)	
H6	−0.4610	0.6856	0.5373	0.066*	
N1	0.2691 (4)	0.54817 (9)	0.1113 (2)	0.0413 (5)	
N2	−0.2076 (4)	0.69473 (10)	0.1842 (2)	0.0473 (5)	
N3	−0.1848 (5)	0.60809 (12)	0.4829 (3)	0.0585 (6)	
O1	0.1028 (3)	0.61679 (8)	0.07990 (19)	0.0447 (5)	

### Atomic displacement parameters (Å^2^)

**Table:**

	*U*^11^	*U*^22^	*U*^33^	*U*^12^	*U*^13^	*U*^23^
C1	0.0409 (14)	0.0379 (12)	0.0351 (12)	−0.0026 (9)	0.0053 (10)	−0.0015 (9)
C2	0.0700 (18)	0.0561 (15)	0.0443 (14)	0.0184 (13)	0.0213 (12)	0.0095 (11)
C3	0.0357 (13)	0.0368 (12)	0.0416 (13)	−0.0028 (10)	0.0084 (10)	−0.0026 (9)
C4	0.0509 (16)	0.0531 (14)	0.0453 (14)	0.0092 (11)	0.0116 (11)	0.0036 (11)
C5	0.0495 (15)	0.0371 (13)	0.0691 (18)	0.0028 (11)	0.0208 (13)	−0.0080 (11)
C6	0.0572 (17)	0.0602 (16)	0.0514 (16)	0.0023 (13)	0.0209 (12)	−0.0122 (12)
N1	0.0429 (12)	0.0382 (10)	0.0439 (11)	0.0056 (8)	0.0100 (9)	0.0015 (8)
N2	0.0504 (13)	0.0348 (11)	0.0594 (13)	0.0017 (9)	0.0176 (10)	0.0011 (8)
N3	0.0575 (14)	0.0742 (14)	0.0477 (13)	0.0163 (12)	0.0207 (10)	0.0036 (10)
O1	0.0499 (10)	0.0432 (9)	0.0441 (9)	0.0108 (7)	0.0175 (7)	0.0044 (6)

### Geometric parameters (Å, °)

**Table:**

C1—N1	1.275 (3)	C4—N3	1.328 (3)
C1—C1^i^	1.478 (4)	C4—H4	0.9300
C1—C2	1.496 (3)	C5—N2	1.336 (3)
C2—H2A	0.9600	C5—C6	1.369 (3)
C2—H2B	0.9600	C5—H5	0.9300
C2—H2C	0.9600	C6—N3	1.325 (3)
C3—N2	1.314 (3)	C6—H6	0.9300
C3—O1	1.376 (3)	N1—O1	1.418 (2)
C3—C4	1.381 (3)		
			
N1—C1—C1^i^	113.6 (2)	N3—C4—H4	119.6
N1—C1—C2	125.36 (19)	C3—C4—H4	119.6
C1^i^—C1—C2	121.1 (2)	N2—C5—C6	122.5 (2)
C1—C2—H2A	109.5	N2—C5—H5	118.8
C1—C2—H2B	109.5	C6—C5—H5	118.8
H2A—C2—H2B	109.5	N3—C6—C5	121.6 (2)
C1—C2—H2C	109.5	N3—C6—H6	119.2
H2A—C2—H2C	109.5	C5—C6—H6	119.2
H2B—C2—H2C	109.5	C1—N1—O1	110.30 (17)
N2—C3—O1	112.01 (18)	C3—N2—C5	115.3 (2)
N2—C3—C4	123.1 (2)	C6—N3—C4	116.7 (2)
O1—C3—C4	124.90 (19)	C3—O1—N1	111.15 (15)
N3—C4—C3	120.9 (2)		
			
N2—C3—C4—N3	0.3 (4)	C6—C5—N2—C3	−0.2 (3)
O1—C3—C4—N3	−179.5 (2)	C5—C6—N3—C4	−0.3 (4)
N2—C5—C6—N3	0.5 (4)	C3—C4—N3—C6	0.0 (3)
C1^i^—C1—N1—O1	−179.78 (19)	N2—C3—O1—N1	−178.19 (15)
C2—C1—N1—O1	0.5 (3)	C4—C3—O1—N1	1.6 (3)
O1—C3—N2—C5	179.60 (18)	C1—N1—O1—C3	−178.45 (17)
C4—C3—N2—C5	−0.2 (3)		

Symmetry codes: (i) −*x*+1, −*y*+1, −*z*.

## References

[bb1] Bruker (2007). *SMART* and *SAINT* Bruker AXS Inc., Madison, Wisconsin, USA.

[bb2] Chen, J. N. & Yang, L. Y. (2008). *Acta Cryst.* E**64**, o1862. 10.1107/S1600536808027128 PMC2960616 21201831

[bb3] Sheldrick, G. M. (1996). *SADABS*, University of Göttingen, Germany.

[bb4] Sheldrick, G. M. (2008). *Acta Cryst.* A**64**, 112–122. 10.1107/S0108767307043930 18156677

